# Food Web Structure in a Harsh Glacier-Fed River

**DOI:** 10.1371/journal.pone.0060899

**Published:** 2013-04-15

**Authors:** Leonie R. Clitherow, Jonathan L. Carrivick, Lee E. Brown

**Affiliations:** 1 Faculty of Biological Science/water@leeds, University of Leeds, Leeds, United Kingdom; 2 School of Geography/water@leeds, University of Leeds, Leeds, United Kingdom; University of Fribourg, Switzerland

## Abstract

Glacier retreat is occurring across the world, and associated river ecosystems are expected to respond more rapidly than those in flowing waters in other regions. The river environment directly downstream of a glacier snout is characterised by extreme low water temperature and unstable channel sediments but these habitats may become rarer with widespread glacier retreat. In these extreme environments food web dynamics have been little studied, yet they could offer opportunities to test food web theories using highly resolved food webs owing to their low taxonomic richness. This study examined the interactions of macroinvertebrate and diatom taxa in the Ödenwinkelkees river, Austrian central Alps between 2006 and 2011. The webs were characterised by low taxon richness (13–22), highly connected individuals (directed connectance up to 0.19) and short mean food chain length (2.00–2.36). The dominant macroinvertebrates were members of the Chironomidae genus *Diamesa* and had an omnivorous diet rich in detritus and diatoms as well as other Chironomidae. Simuliidae (typically detritivorous filterers) had a diet rich in diatoms but also showed evidence of predation on Chironomidae larvae. Food webs showed strong species-averaged and individual size structuring but mass-abundance scaling coefficients were larger than those predicted by metabolic theory, perhaps due to a combination of spatial averaging effects of patchily distributed consumers and resources, and/or consumers deriving unquantified resources from microorganisms attached to the large amounts of ingested rock fragments. Comparison of food web structural metrics with those from 62 published river webs suggest these glacier-fed river food web properties were extreme but in line with general food web scaling predictions, a finding which could prove useful to forecast the effects of anticipated future glacier retreat on the structure of aquatic food webs.

## Introduction

One of the main organisational components in an ecosystem is the food web, a description of ‘who-eats-whom’ [1], [2]. The study of food webs has increased exponentially since the 1970’s and detailed examination of whole food web interactions has been acknowledged as key to understanding the effects of habitat change because emergent properties in complex systems can undermine predictions from responses seen at lower levels of organisation [3]–[6]. Knowledge of the many feeding linkages within an ecosystem is vital to comprehend how a community of individuals can persist over time, as well as describing its stability with respect to environmental or biological perturbations [2], [3]. This is of particular importance in terms of climatic change which is likely to alter hydrological and thermal regimes markedly, leading to major changes in aquatic food webs [5]–[7].

Climate change threatens high latitude and mountainous areas, and warming is occurring more rapidly in many of these regions than anywhere else [8], [9]. Increased attention is being paid to alpine rivers as their fragility and vulnerability to climate change becomes more apparent [10]–[13]. Glaciers in these environments are retreating and downwasting rapidly, changing river flow and thermal regimes, fluvial geomorphology and water chemistry [14]. In turn, benthic communities in glacial rivers are seriously at risk because they are deterministically influenced by meltwater from glaciers and snowpacks, and there are limited options for dispersal [13], [15]. If glacial melt water inputs are reduced, or lost entirely, some macroinvertebrate species may become extinct [13], [16], with largely unknown direct and indirect consequences for ecosystem structure and functioning via alterations to species interactions [14], [17].

Rivers immediately downstream of glacier outlets represent a unique harsh habitat for aquatic communities [18]. Glacial rivers are characterised by year round water temperature typically ≤2°C, seasonal peaks in discharge (typically July in the Northern hemisphere), high flows in the afternoon due to diel melt patterns, highly unstable channel morphology and high turbidity from suspended glacial sediment [19], [20]. The conditions typical of glacier-fed streams mean few macroinvertebrate species are found in these areas [10]. The chironomid genus *Diamesa* is often found in glacier-fed streams as well as some Simuliidae [11], [16], [19], [21]–[23]. *Diamesa latitarsis* grp. often dominate rivers in the Alps and other European mountain rivers where water temperature does not exceed 2°C [16], [21], [24]. Substratum instability, suspended solid concentration, and channel width:depth ratio typically decrease downstream from the glacier and lead to increased macroinvertebrate and algal taxonomic richness and total abundance [18], [24], [25], indicating a reduction in habitat harshness.

Allochthonous organic inputs are typically low in glacial streams due to the lack of bankside vegetation and primary production is therefore of greater importance [10], [17], [21]. Aquatic flora tends to be dominated by diatoms, cyanobacteria and the golden algae *Hydrurus foetidus* [25], [26]. What is unclear is the nature of interactions between macroinvertebrate consumers and these basal resources. Limited gut content analysis of mayflies and stoneflies (Ephemeroptera and Plecoptera) in glacial rivers has suggested a dominance of omnivory with detritus, algae and diatoms all present [10]. However, despite the current level of research on aquatic connectance food webs (e.g. [27]–[30]), including some in mountain areas [31], [32], no connectance food webs have yet been constructed for glacier-fed river communities. This prevents a more complete understanding of the networks of feeding interactions which are fundamental to the dynamic stability of whole food webs [33].

Food web studies undertaken to date in alpine glacier-fed rivers have favoured stable isotope methods (e.g. [10], [17]), preventing analyses on the network of individual feeding interactions. In harsh environments with low rates of primary production, minimal allochthonous organic matter inputs and low predation, food webs should be dominated by invertebrates with generalist feeding habits (e.g. [34]), leading to high connectance. While freshwater food webs are generally strongly structured according to the body size of constituent organisms [35], the harsh habitat of glacial rivers is known to restrict the abundance of large bodied organisms (e.g. [15], [36]) The consequences of this restricted size spectrum in glacial systems are unknown, although it is likely that mean and maximum food chain lengths will be short [37], [38] owing to the preponderance of feeding links from diatoms/algae to Chironomidae larvae, and lack of predators, respectively. Moreover, some recent food web studies examining mass (*M*) versus abundance (*N*) scaling have suggested that metabolic theory predictions of coefficients ranging between −3/4 to −1 [39] may not be observed in streams due to an abundance of allochthonous detritus subsidising secondary production [30], [40]. While these predictions have not been tested for alpine rivers, the notable lack of allochthonous detrital inputs in these environments could underpin differences in food web allometric properties.

This study aimed to: (1) characterise the trophic links of a glacier-fed river in the Austrian central Alps; (2) quantify food web connectance and size structure properties, and; (3) contextualise these characteristics against published literature for other lotic systems. We hypothesised that: (H_1_) diatoms and detritus would be the dominant food source for the macroinvertebrates found in the glacial stream; (H_2_) the restricted producer base and dominance of non-predatory invertebrate groups (Diamesinae, Simuliidae) would mean food chain lengths were short (i.e. two trophic components), but (H_3_) the prevalence of omnivory would mean links per species and connectance (the proportion of all possible links realised) would be high, but characteristic path lengths would be low. Finally, (H_4_), we expected that *MN* scaling coefficients would be close to −1 as predicted by metabolic theory for webs with multiple trophic levels (e.g. [39]), owing to the predominant autochthonous resource base in alpine zone rivers.

## Study Site and Methods

**Figure 1:**
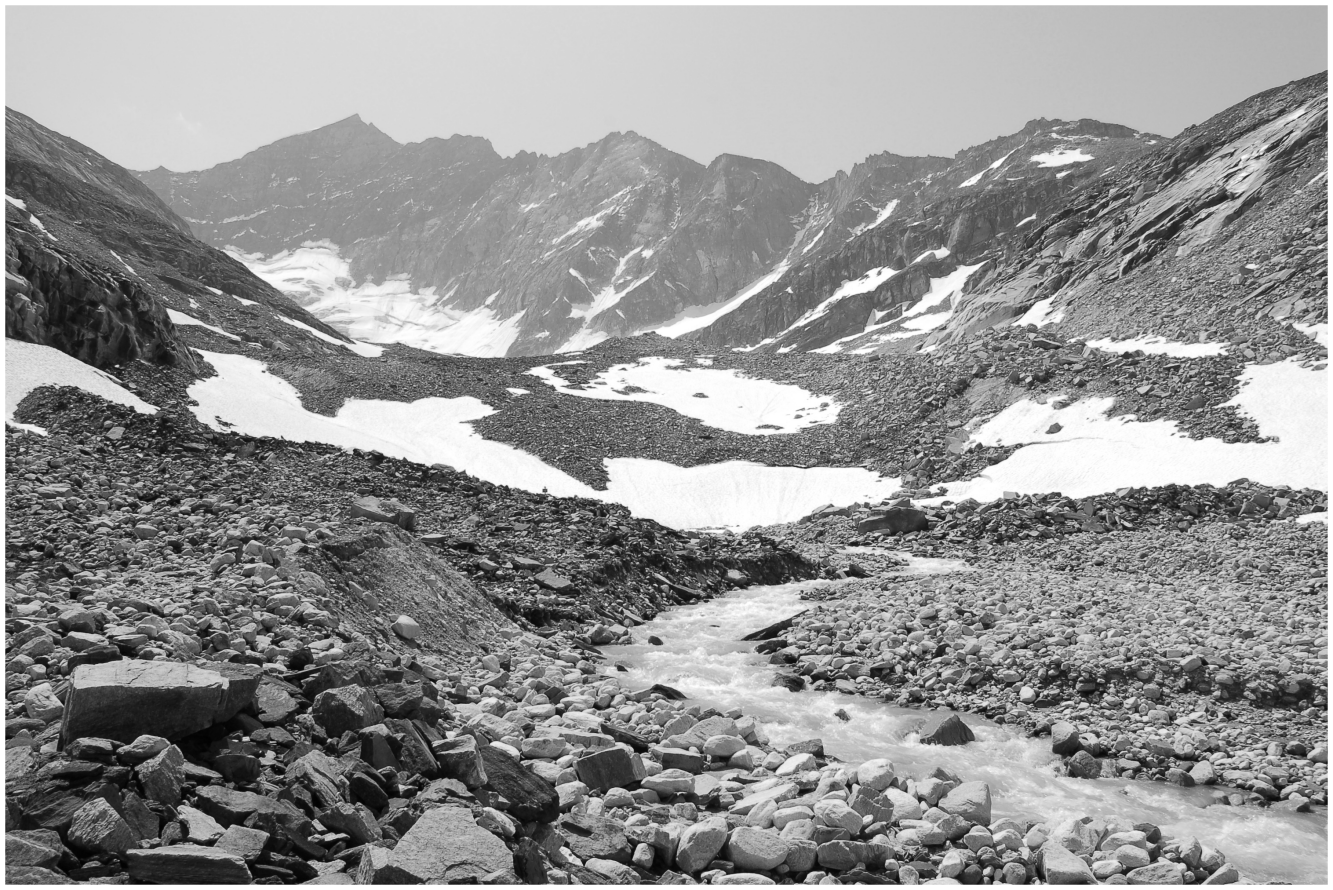
Ödenwinkelkees river sampling site. Stream width in the foreground is approximately 5 m.

Samples were collected from the Ödenwinkelkees river, Hohe Tauern National Park (47°08′N, 12°38′E), Central Austrian Alps, in July 2006, 2008 and 2011. All necessary permits were obtained for the described field studies from the Hohe Tauern National Park Authorities. The Ödenwinkelkees has retreated at a mean rate of ∼10 m yr^−1^ since its maximum extent in 1850 [41], yet despite this, 19.5% of the catchment remains glaciated [42]. Annual mean air temperature is −0.3°C and annual mean precipitation is 2397 mm [41]. The samples collected for this study originated from directly below the Ödenwinkelkees snout (Figure 1), at around 2194 m a.s.l. Maximum water temperature of the river at the sampling location is consistently <2°C (Table 1; [43]). The glacial valley is north facing and steep sided (30–60°) with predominantly bedrock sides, although some thin soils and vegetation exist down-valley of the glacier [42].

**Table 1:** Food web summary statistics (* denotes chain length statistics incorporating cannibalistic links).

	2006	2008	2011	Composite
Water temperature (°C)	0.8	0.7	1.8	–
***Connectance web statistics***				
No. taxa (*S*)	13	19	19	23
No. links (*L*)	16	51	67	85
Links per species (*L/S*)	1.23	2.68	3.53	3.70
Directed connectance (*C*)	0.05	0.14	0.19	0.16
Characteristic path length (*D*)	2.26	1.95	1.76	1.86
Mean chain length	2.0	2.36	2.27	2.28
Max chain length	2.0	3.0	3.0	3.0
Mean chain length*	2.0	2.68	2.61	2.53
Max chain length*	2.0	4.0	4.0	4.0
% predator	0	16	16	13
% primary consumer	38	26	32	35
% basal	62	58	53	52
***Tritrophic web statistics***				
Number of food chains	–	–	57	–
Number of three nodefood chains	–	–	14	–
Community span	–	–	13.12	–
Mean (median) link angle	–	–	−27.74 (−25.81)	–
Mean (median) link length	–	–	8.73 (8.96)	–
Mean (median) L_upper_	–	–	1.16 (1.16)	–
Mean (median) L_lower_	–	–	8.49 (8.28)	–
Mean (median) A_upper_	–	–	−19.33 (−19.33)	–
Mean (median) A_lower_	–	–	−23.03 (−22.66)	–
Mean (median) A_between_	–	–	3.70 (4.82)	–
Mean (median) 2-span	–	–	9.65 (9.69)	–
Mean (median) countchain length	–	–	1.29 (1.0)	–
Mean (median) sumchain lengths	–	–	9.35 (9.24)	–
Mean (median) chain span	–	–	9.35 (9.24)	–

[L and A are the length and angle, respectively, of a link between consumer and resource in three node food chains; _upper_ refers to the link between a consumer and intermediate taxon (resource); _lower_ refers to the link between an intermediate taxon (consumer) and a resource; A_between_ refers to the angle between the upper and lower components]. Tritrophic measures are provided for 2011 only because these require additional estimates of body mass and abundance which were not available for all taxa in 2006 and 2008.

Macroinvertebrate samples were collected in each year of sampling by disturbing ten 0.1 m^2^ areas of riverbed using a Surber sampler with a 250 µm mesh net. Additional macroinvertebrates for gut content analysis were picked from rocks with fine forceps. Samples were preserved in the field in 70% ethanol. Benthic algal samples were collected by randomly selecting 10 cobbles, scrubbing the upper surface with a stiff toothbrush, rinsing in 60 ml of deionised water and then freezing the resulting suspension. Rock surface area was calculated by tracing outlines onto acetate sheets, cutting out and weighing, then scaling relative to the weight of a 5×5 cm piece of acetate. In the laboratory, algal scrubs were centrifuged then deposits re-suspended in 5 ml of deionised water and cleaned of organic material using 20 ml of H_2_O_2_ while heating to 90°C for approximately 35 min. Remaining H_2_O_2_ and carbonates were subsequently removed using 50% HCl. Samples were then washed with deionised water and centrifuged again a further three times. Finally, for each sample, 0.5 ml of suspension was pipetted onto glass slides and allowed to settle before being mounted in Naphrax.

Feeding linkages were determined by direct observation of gut contents at ×1000 magnification. Macroinvertebrate guts were mounted on to microscope slides by removing the foregut following dissection of the invertebrate. Contents were mounted in Euparol and secured with a cover slip. The remains of Chironomidae and Simuliidae (head capsule and body) were subsequently cleared with 10% KOH. After 24 hours, invertebrates were cleaned with distilled water before being placed in glacial acetic acid. Finally the invertebrates were mounted on to a slide (ventral side up) with Euparol [44]. The samples were then identified to the lowest possible taxonomic level under a high-power light microscope (×200–1000 magnification) with reference to [45]–[47]. Head capsule width, body length, antenna length and mandible length were measured with an eye piece graticule to the nearest 0.01 mm. Gut contents and benthic diatom samples were identified to the lowest practicable taxonomic level using appropriate keys and previous alpine stream work [48]–[53]. Ingested prey items were measured to the nearest 0.001 mm using an eye piece graticule. Yield-effort curves were constructed for each food web to aid interpretation of food web properties (Figure S1).

For a small number of ingested invertebrates that were partly digested, measurements of either head capsule width, antenna length or mandible length were converted to body length estimates using regression from measurements made on whole individuals collected from the benthos. Body length of macroinvertebrates and their ingested prey were converted to units of dry mass (mg) using a variety of published length–mass regressions [54], [55]. Diatom mass was estimated from length, width and depth measurements of individual frustules, which were converted to biovolume using published equations [56], [57]. Biovolumes were subsequently converted to a mass of carbon [58], before converting to dry mass based on an average carbon content of 19% for freshwater diatoms [59]. Food items identified via gut content analysis were recorded in a matrix as presence/absence. Abundance of ingested items was not calculated by counting individual food items because it could not be guaranteed that entire gut contents were mounted on to slides (particularly for extremely small individuals). Additionally, previous work has suggested that some chironomid larvae may partially regurgitate gut contents during preservation (e.g. [60]).

### Data Analysis

Separate food webs were constructed for each year of sampling and a fourth ‘composite’ web was constructed by pooling all food web data from each year of study. Food web images were produced with FoodWeb3D [61]. Taxa were classified as either basal (i.e. producers), primary consumers (those consuming only basal taxa), or predators (those consuming invertebrates). Summary statistics for food webs were calculated as detailed in [30] including taxonomic richness of the food web (*S*), number of links between individuals (*L*), linkage density (*L/S*) and directed connectance (*C* = *L/S* ^2^). Food chain lengths were calculated from the number of trophic elements in each individual food chain (i.e. consumer *a* eating only resource *x* = chain length of 2; ref [62]). Comparisons with other studies that count only the number of links in a chain (i.e. consumer *a* eating only resource *x* = chain length of 1) can be made by subtracting 1 from reported values, if published studies explicitly state the method that has been adopted. Trophic height was calculated as 1+ mean trophic height of a consumer’s resources [63]. Additionally, path lengths (the fewest links connecting each pair of taxa; *d*), the mean of which (for the community) is the characteristic path length (*D*), were determined using Pajek v1.23 [64], [65] and used to test the two degrees of separation theory of Williams *et al*. [66]. All statistics were compared to published data available for 59 food webs from stream environments as listed in Brown *et al*. [30]. We also incorporated three additional food webs from the non glacier-fed Estaragne stream located in the alpine zone of the French Pyrénées [31]. We analysed the relationship between *S* and *L* for this database and the four new webs using Ordinary Least Squares (OLS) linear regression, to assess whether the Ödenwinkelkees river food webs could be considered as ‘extreme’ cases of stream food web structure or whether they possessed unique properties.

To determine the extent to which the Ödenwinkelkees river food webs displayed the non-random organization seen in many natural food webs [67], regression was used to fit models (exponential, power, linear) to cumulative degree distributions, with the model producing the highest R^2^ being retained. OLS linear regression was used to describe allometric scaling relationships between individual consumer and resource mass, Log_10_ *M* and trophic height, and Log_10_ *M* and Log_10_ *N* for the 2011 food web. Detritus particles, which had no measurable *M* and *N,* and some taxa which were found in guts but not in the benthos (i.e. *N* unavailable), were excluded from *MN* analyses. Both species-averaged data, and individual size data irrespective of species identity, were used to assess *MN* relationships. For the latter, individuals were first assigned to log_2_ integer size bins, and the mid-point of each bin was log_10_ transformed and used thereafter [30], [68]. *N* was divided by the width of each body mass size class to calculate normalized abundance (N*; ref [69]). The scaling coefficient of the subsequent MN* regression +1 was used to compare empirical measures with the −3/4 to −1 scaling predictions of metabolic theory [39], [68]. For comparison with the approach used by O’Gorman *et al*. [70], we repeated the analysis with individuals assigned to log_10_ size bins. Exponents of −3/4 are predicted for a single trophic level where species use the same resource and metabolism scales with body mass, while −1 is predicted for several trophic levels with particular assimilation efficiencies and predator:prey mass ratios [39]. All regression analyses were undertaken using SPSS 15.0 (IBM Corporation, Somers, NY, USA) and considered significant where *P*<0.05. Additionally, we used Cheddar v0.1–616 [71] to calculate the suite of tritrophic food web statistics that can be derived from (i) trophic link lengths between consumers and resources (i.e. n orders of magnitude difference in *M*+*N*); (ii) trophic link angles from resources to consumers, which are a function of rate of change in biomass, population productivity and population consumption (for full details see refs [72]–[74]).

## Results

The total number of taxa recorded at the study site was 22, of which 11 were diatoms and 11 were invertebrates (including eight Chironomidae [seven Diamesinae, one Orthocladiinae], one Ephemeroptera [*Baetis*], one Trichoptera [Limnephilidae] and one Simuliidae [*Prosimulium*]). Total macroinvertebrate abundance was low but highly patchy, with an average 19 individuals per m^2^ for Chironomidae and only one individual per m^2^ for *Prosimulium*. *Baetis* and Limnephilidae were found only as isolated individuals in hand-picked samples. *Encyonema* sp. was the most abundant diatom, with *Diatoma mesodon* and *Gomphonema* sp. A (see Table S1) also relatively abundant; generally the group were distributed patchily with maximum abundance of ∼81×10^3^ m^−2^ but typically <10×10^3^ m^−2^. In total, 144 guts were examined, producing 314 observations of consumer-resource interactions. All guts were dominated volumetrically by rock fragments with small quantities of organic detritus; 66 contained diatoms and nine contained remains of invertebrate prey. Of the 22 taxa, 52% were basal taxa, 35% primary consumers and 13% predators (Table 1). *Diamesa latitarsis/steinboecki* were cannibalistic, while *Diamesa* spp. and Orthocladiinae were found in the guts of *Prosimuliium*.

**Figure 2:**
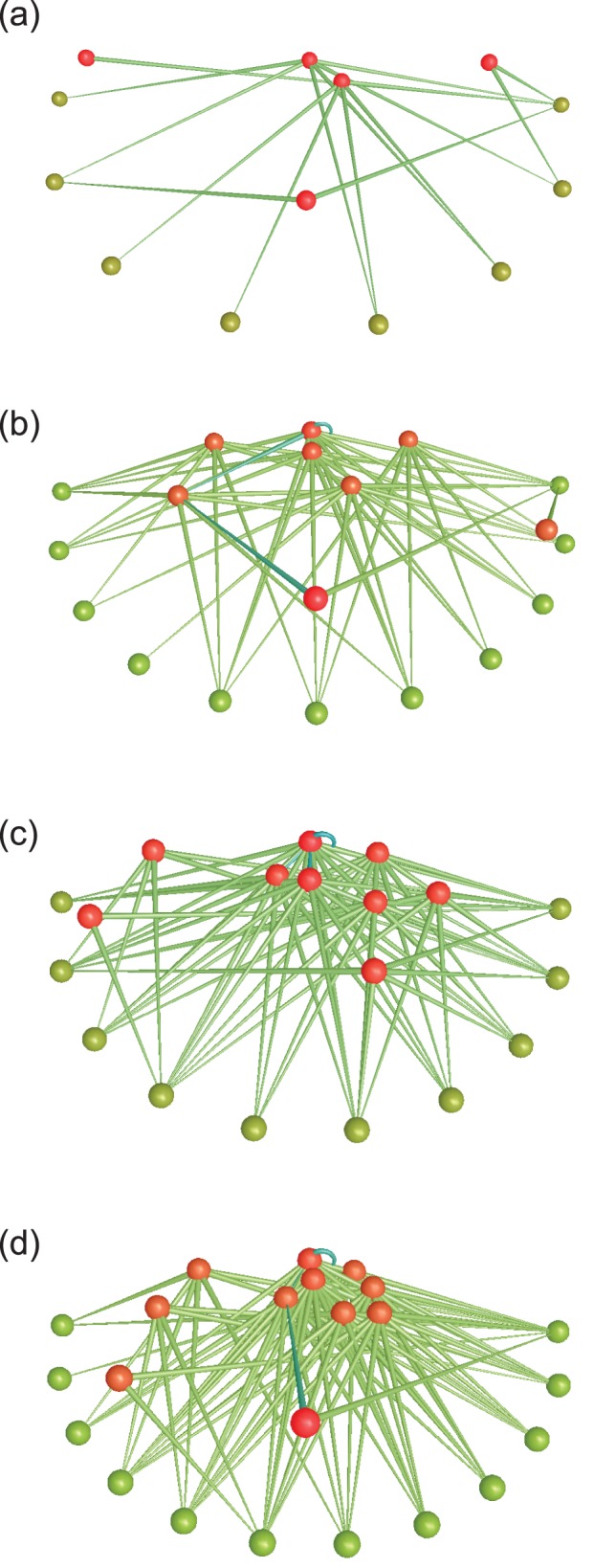
Food webs collected in Ödenwinkelkees river. (a) 2006, (b) 2008, (c) 2011 and (d) composite of the three webs. Green nodes represent primary producers, red nodes represent consumers. Green lines represent feeding links and blue lines represent cannibalistic links. Food web diagrams created using FoodWeb3D [61].

**Figure 3:**
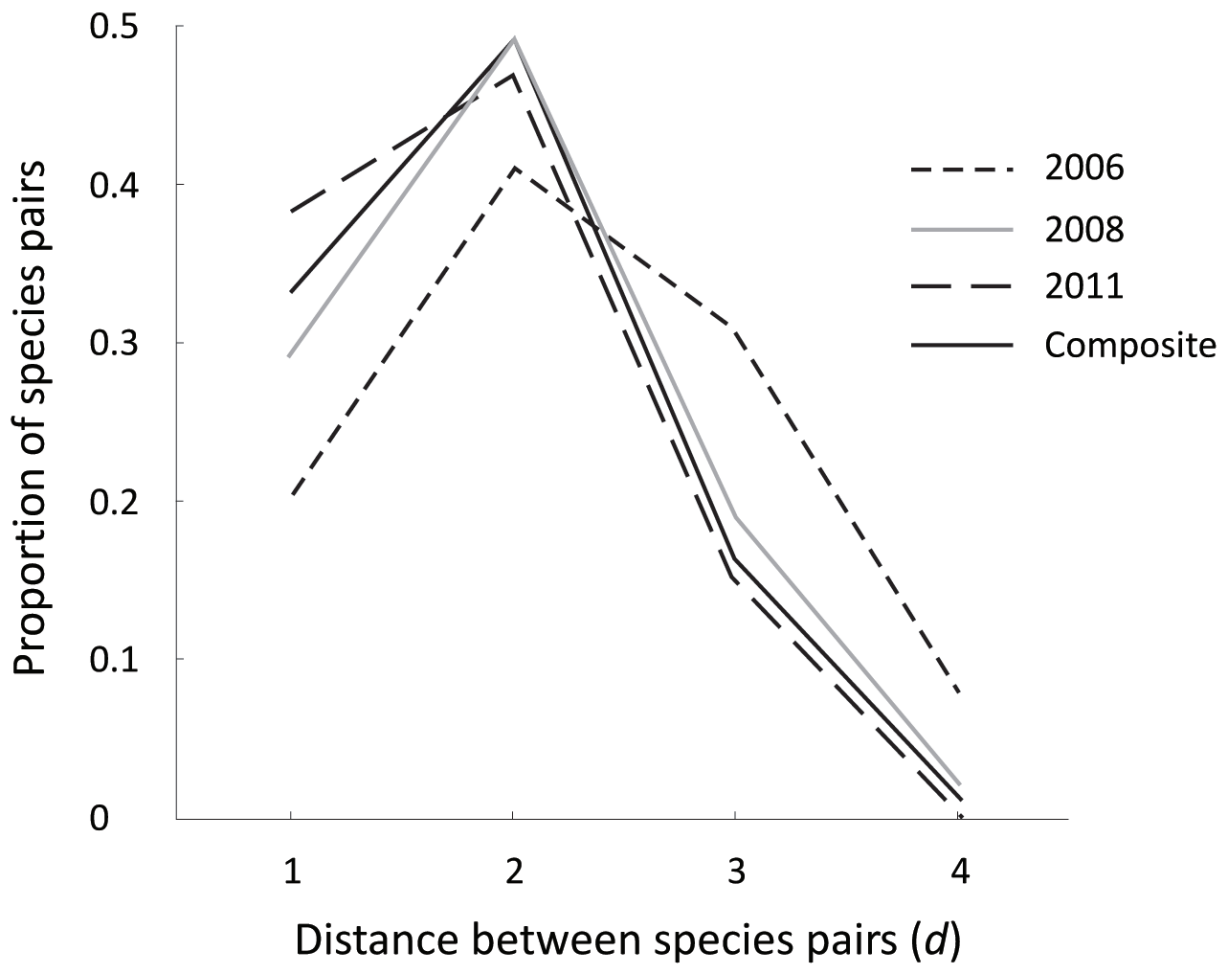
Path length (d) distributions for the four food webs. Path lengths were calculated as the fewest links connecting each pair of taxa, measured for each pair of nodes.

The number of taxa (*S*) was the same in 2008 and 2011, but clearly lower in 2006 (Table 1; Figure 2). The number of links (*L*) in each food web varied per year but 2006 was an obvious outlier; yield-effort curves (Figure S1) also suggested this web was incompletely described. Links per species in 2008 and 2011 were 2.68 and 3.53 respectively. Directed connectance (*C*) was lowest for 2006 and highest in 2011 (Table 1). The composite web (Table S1) of 22 taxa (plus detritus) had 85 links, with 3.70 links per species and a directed connectance of 0.16. Characteristic path lengths were typically <2, and analysis of individual path length distances indicated that >92% of taxa recorded in the Ödenwinkelkees river were connected by only one or two trophic links (Figure 3). Mean chain length (MCL) was similar between years, ranging from 2.00 to 2.36.

**Figure 4:**
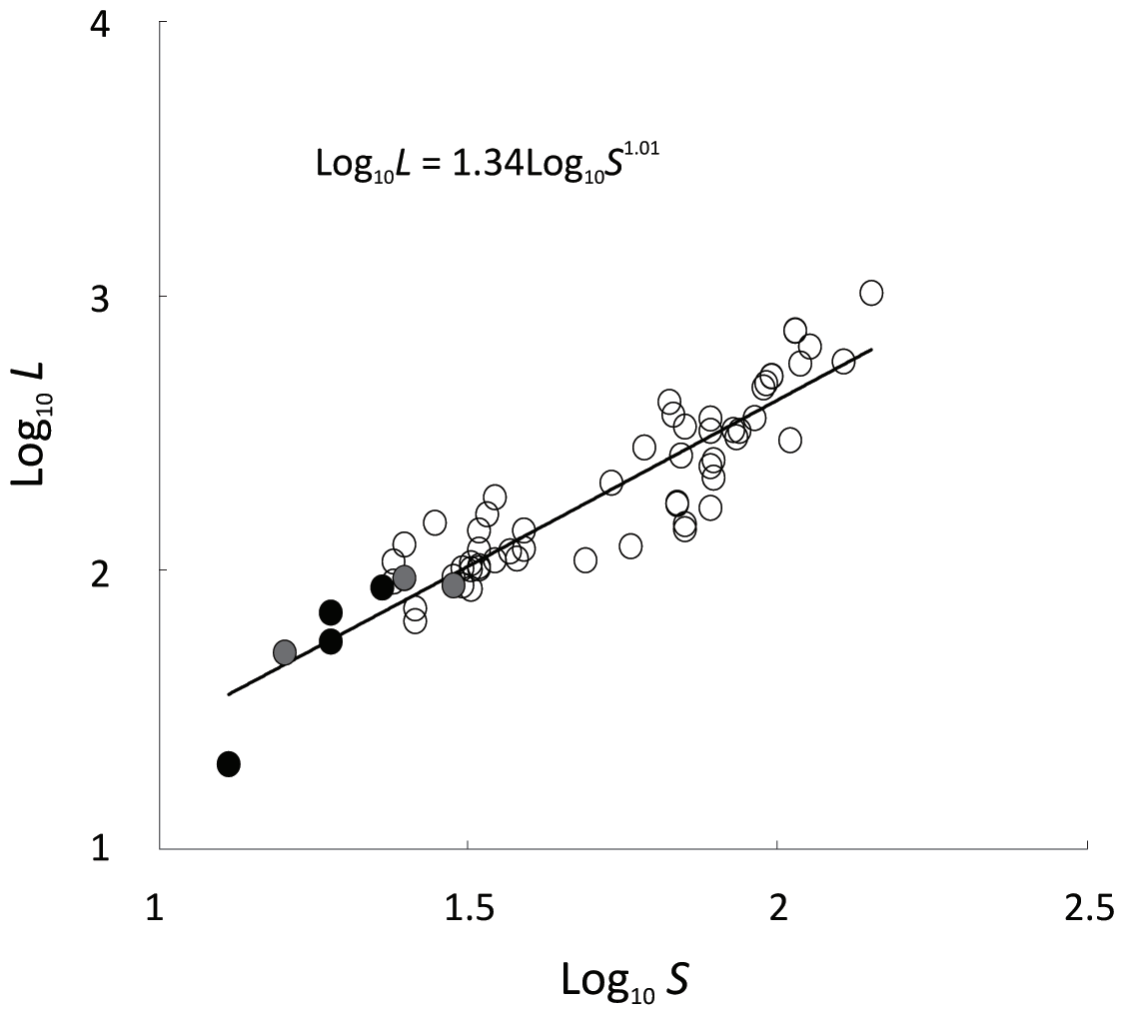
Relationship between taxon richness (*S*) and number of links (*L*). Ödenwinkelkees food webs are shown in black and reported literature values as open circles. Grey symbols highlight the alpine stream food webs collected by Lavandier & Décamps [31].

The Ödenwinkelkees river data were compared to food web statistics gathered from 62 published food webs from lotic systems. The mean *S* in these webs (62; range: 16–142) and mean number of links (305; range: 62–1383) were far in excess of those observed in the Ödenwinkelkees river. The overall relationship between *S* and *L* was strong and positive (R^2^ = 0.716, *P*<0.001; Figure 4) although the 2006 food web appeared as a clear outlier from the other webs (Figure 4). *S* and *L* were the lowest of any of the published food webs except for one mountain stream in the French Pyrénées (Figure 4; Table S2). Connectance in the Alpine food webs was consistently higher than reported for food webs in other studies, which had a mean connectance of 0.10 (±0.07). MCL was shorter than most reported food webs.

**Figure 5:**
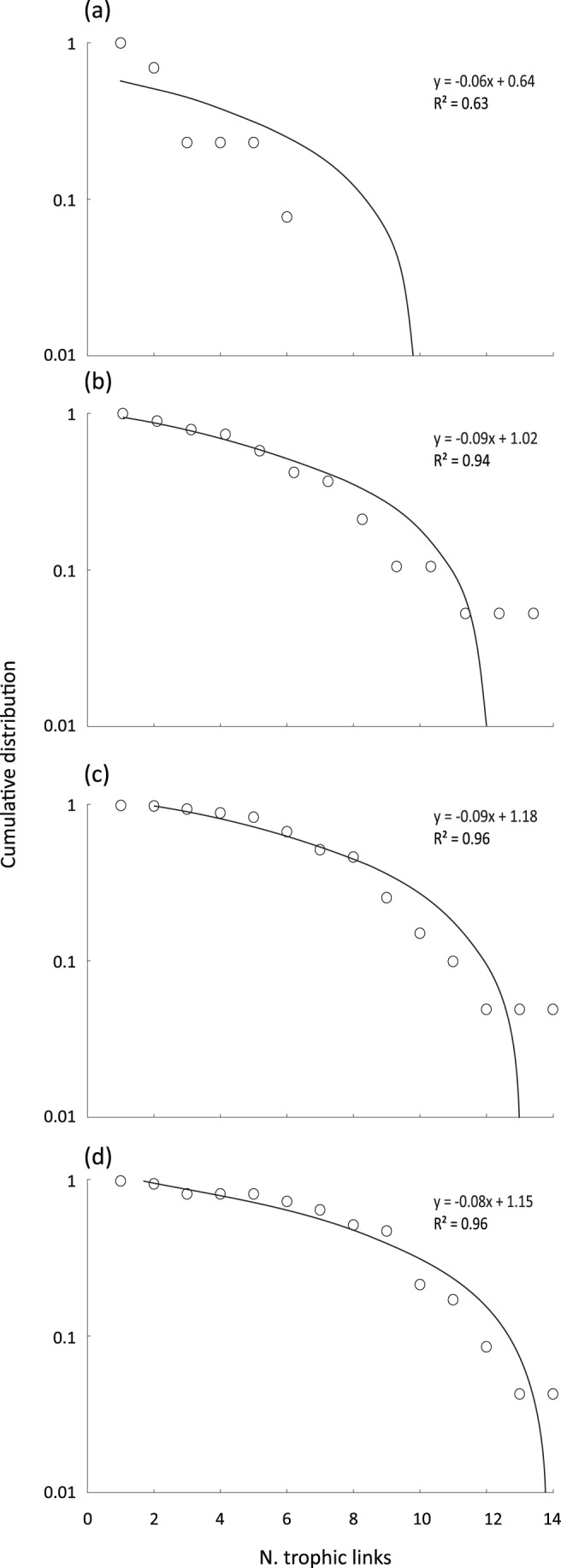
Cumulative distribution of trophic links per species for the four food webs. Y-axis indicates number of trophic links. All linear relationships *P*<0.05.

**Figure 6:**
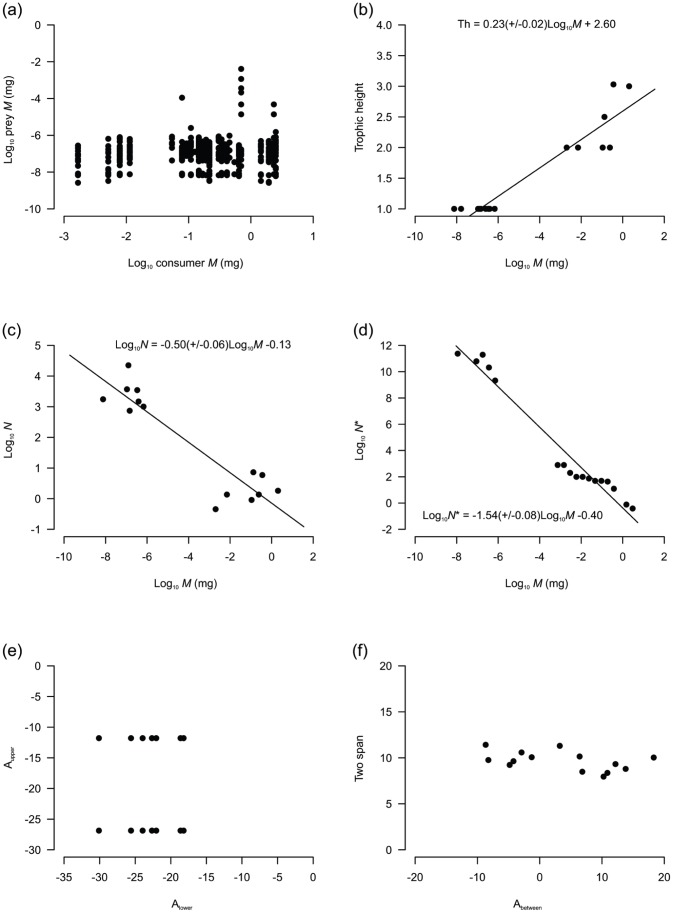
Food web body size relationships derived from the 2011 food web. (a) relationship between body mass of individual consumers and resources; (b) relationship between body mass of taxon and trophic height; (c) relationship between species averaged body mass (*M*) and abundance (*N*); (d) relationship between individual *M* and N* (normalized abundance, see methods). (Error terms in parentheses for b and c denote 95% confidence intervals; NB. Data shown for log_2_ integer approach only); (e) upper angle (A_upper_) versus lower angle (A_lower_) of all three- node chains within food webs; (f) two-span as a function of between angle (A_between_) in three node chains. See Table 1 caption for definition of these measures.

Cumulative degree distributions for all four webs were described best by linear functions (R^2^>0.94 with the exception of the 2006 web; Figure 5). Body mass spanned 9 orders of magnitude for the 2011 Ödenwinkelkees food web. There was no clear relationship between individual consumer and resource mass (Figure 6a) but trophic height was strongly related to taxon averaged body mass (Figure 6b). *MN* relationships were described by statistically significant linear regressions (R^2^ = 0.84 for species averaged data, R^2^ = 0.96 for individual data; *P*<0.001 for both regressions; Figure 6c and d). Slope was −0.50 for species-averaged data, and for the individual size distribution data slope was −0.54 using the log_2_ approach and −0.42 for the log_10_ approach. Link angles and between angles were tightly constrained in the food web (Figure 6e and f; Table 1).

## Discussion

The diatom flora was relatively restricted containing just 11 taxa, although similar findings have been seen in glacier-fed rivers elsewhere in the Alps [26]. Common benthic diatom species observed in the benthos and invertebrate gut contents included *Encyonema* sp., *Acthnanthes* spp., *Diatoma mesodon* and *Gomphonema micropus* which are common in high altitude European rivers [25]. Some diatoms could not be identified further than genus due to their small size restricting the visibility of morphological features (see [53]). Without further detailed study of the diatoms using, for example, electron microscopy, precise species identification cannot be certain. Further studies in the area focusing on detailed diatom species identification (*cf*. [48]) would be particularly useful to increase the knowledge of the basal resources available and thus improve the taxonomic resolution of alpine food web studies.

The macroinvertebrate assemblage in the Ödenwinkelkees river was dominated by the chironomid genus *Diamesa*, which is typical of cold streams in Europe [16], [19], [21]–[23]. A large proportion of the individuals were identified as *D. latitarsis/steinboecki* because they possessed particularly short anal setae (*cf*. *D. steinboecki*) but four were present instead of three (*cf*. *D. latitarsis*). However, other individuals that could definitely be identified as *D. latitarsis* appeared morphologically different to *D. latitarsis/steinboecki*, with longer anal setae and slightly different mentum shapes. At least two subspecies of *D. latitarsis* were likely present in the Ödenwinkelkees river necessitating further taxonomic work to differentiate them. Other invertebrate taxa identified from close to the glacier snout included *Baetis,* Limnephilidae and *Prosimulium*, which are typically expected only from glacier streams much further from the ice margin where water temperature and channel stability are higher (e.g. [18]). The presence of these non Diptera taxa at <2°C may indicate either some local adaptation, or a wide array of phenotype plasticity allowing colonisation of the glacier-fed river at various locations (*cf.* work from New Zealand, which indicates the presence of mayflies at low water temperatures, [75]). Studies in other European glacier-fed streams have found similar taxon assemblages directly downstream of the glacier snout, namely: *D. latitarsis* grp., *D. steinboecki*, and *D. cinerella/zernyi*, although EPT taxa (Ephemeroptera, Plecoptera, Trichoptera) were only found at least 200 m from the glacier snout [24], [76], [77]. None of these studies, however, reported any taxa resembling *D. latitarsis/steinboecki*.

### Food Web Structure

The Ödenwinkelkees river food webs were dominated by links from consumers to detritus and diatoms; supporting H_1_ (diatoms and detritus would be the dominant food source for macroinvertebrates). While all consumers had evidence of detritus in their guts, absolute quantities were very small reflecting the lack of riparian allochthonous organic matter subsidies to these systems, similar to findings of Zah *et al*. [17] where detritus was only considered important in groundwater fed alpine stream food webs. While diatoms were more abundant in the diet than detritus fragments, all guts were full of fine glacial rock fragments, far more so than observed by Füreder *et al*. [22]. The reason for this is unclear but could be either unintentional uptake whilst grazing algae or collecting detrital resources from rock surfaces, or could be an adaptive mechanism to enable consumption of microorganisms which adsorb to abundant rock surfaces in glacial environments [78], [79].

Compared with the majority of food web studies undertaken in temperate zone rivers [30], [80], taxon richness within the Ödenwinkelkees river was consistently lower than that found in almost all other food web studies, due to the harsh glacier-fed river. Only one published web (from the French Pyrénées) had similarly low numbers of *S* and *L* but this web had low taxonomic resolution of basal taxa [31] and is therefore not directly comparable to our webs where we attempted to resolve the diatoms as far as possible. The four Ödenwinkelkees webs were at the extreme low end of published estimates for *S* and *L*; in particular, the food web for 2006 was the smallest of the four constructed, but this food web was clearly different from those created for 2008 and 2011, and appears to be an incomplete representation. All invertebrates examined in 2006 contained rock fragments and detritus but diatoms were particularly scarce for no clear reason.

The four Ödenwinkelkees webs had some of the shortest food chain lengths of published studies to date. Predatory links amongst *Diamesa* and Simuliidae, which are typically considered grazers/collectors and detritivorous collector/filterers, respectively, caused the MCL to range up to 2.36; we therefore rejected part of H_2_ that chains would be shorter and link only diatoms/detritus and primary consumers (i.e. MCL = 2). However, while the majority of links were between two trophic elements and predatory links were few, shorter mean chain lengths using the trophic element approach have been recorded in a previous mesocosm based study [30]. This was due to a very wide range of basal species and numerous primary consumers but few predators, leading to two trophic element chains dominating. While different approaches to calculating mean chain length are used and often not reported explicitly by authors [81], studies reporting chain lengths <2 must obviously use the approach of counting links rather than trophic elements (e.g. [1], [29], [32]). Chain length for the Ödenwinkelkees webs using the same method generates values from 1.0–1.36, making these the shortest mean chain lengths reported to date from flowing waters, a property which can be related to low primary production and the near absence of allochthonous subsidies (*cf*. [82]) in these rivers.

Despite taxon richness being low in the Ödenwinkelkees river, linkage density was well within the range of the literature values, and connectance (excluding the 2006 food web) was higher than all other studies, even alpine stream studies from the Pyrénées [31], thus we accepted part of H_3_ (that omnivory would lead to high mean links per species and high connectance). Decreasing connectance with food web size has been documented previously [83], [84] and seems to hold true across lotic ecosystems. The largest food webs typically had connectance values of <0.10, compared to 0.14–0.19 in the Ödenwinkelkees river (excluding 2006). The generalist feeding patterns in the Ödenwinkelkees river, whereby many macroinvertebrates consumed detrital matter as well as the majority of the diatoms present (particularly *Encyonema* sp.), increased connectance markedly. While omnivory is relatively common among stream biota [62], some stream invertebrates show specialised feeding patterns; for example Thompson and Townsend [29] found very low connectance in food webs from New Zealand and North America and some taxa were found with only one algal species in their gut contents. The high level of generalist feeding in the Ödenwinkelkees web meant that 92% of taxa in the composite web could be connected by 1 or 2 links, supporting the second part of H_3_ that the two degrees of separation theory [66] would hold in this system. The number of species pairs connected by two or fewer links (92%) was far in excess of the 80% reported by Williams *et al*. [66] and the 71% reported by Brown *et al*. [30].

Cannibalism was observed in some Ödenwinkelkees river food webs but is not unique to glacial streams. Competition for space and resources between individual invertebrates sometimes causes larger instars to consume smaller instars of the same species [28], [62]. This might be the reason why the most common chironomid (*D. latitarsis/steinboecki*) demonstrated cannibalistic feeding links in the Ödenwinkelkees river. During hand searching it was observed that the highest densities of Chironomidae were located on relatively few ‘stable’ boulders at the river’s edge in an otherwise highly unstable environment. In 2011 the most prolific predator was an individual of *D. latitarsis/steinboecki*, which had 24 body parts and seven head capsules of other *Diamesa* (many of which were likely to be the same species) in its gut. This single individual was noticed to be somewhat larger than the others of the same species group which had also ingested invertebrate prey items. However, even this predator still contained several diatom taxa as well as detritus, although it is not clear whether these were ingested directly from the benthos or if they had been consumed previously by prey.

Yield effort curves for diatom identification suggest that sampling effort, particularly in 2011, recorded all the taxa present (Figure S1). However, with the patchy spatial distribution of macroinvertebrates in the Ödenwinkelkees river, it can be difficult to sample sufficient individuals to characterise every possible interaction occurring. For the most abundant *Diamesa* sp., an asymptote for the number of diatom taxa ingested was reached, although this was not always the case for the rarer taxa. This underlines the difficulty of characterising feeding links in harsh river systems where individuals are patchily distributed at low abundance.

### Allometric Scaling

The constituent members of the Ödenwinkelkees food web spanned ∼9 orders of magnitude body size, slightly less than some other stream food webs (e.g. [30], [40]) owing to the dominance of relatively small Chironomidae and Simuliidae larvae at the apex of the food web. Examination of the relationships between individual consumer and prey masses revealed small items (diatoms) were the dominant component of all consumer diets as suggested by Zah *et al*. [17] for a glacial stream community in the Swiss Alps. A broadening of diet breadth with increased body size was evident, but patterns were not as clear as those seen elsewhere (e.g. [85]), and it is notable that the number of individuals with animal prey in their guts was very small in our study. Analysis of greater numbers of consumers is necessary to quantify fully the nature of predatory links in glacial systems (*cf.* [35]), particularly because these macroinvertebrates are not obligate predators and distributions are spatially patchy, so the likelihood of observing these links is low. Trophic height showed an increase with taxon body size consistent with several stream and marine webs analysed by Gilljam *et al*. [63], because larger species feed on resources (i.e. other consumers) higher up in the food web.

Mass-abundance (*MN*) relationships for both taxonomic and individual based datasets revealed scaling coefficients that were significantly higher than those predicted by metabolic theory [39]. This phenomenon has been observed in several other running water food webs [30], [40] and has been attributed to detrital inputs from riparian vegetation and upstream river reaches subsidising secondary production and because detrital resources cannot be represented in *MN* plots. Thus, consumer abundances are higher than would be expected from an autochthonous driven system [74]. However, detrital subsidies to alpine glacier-fed rivers are largely negligible [86] owing to the lack of vegetation in the alpine zone. The higher than expected coefficient therefore appears to be a function of elevated macroinvertebrate or lowered diatom abundances, by approximately two orders of magnitude (or a combination of both). Potential reasons for this discrepancy are that macroinvertebrates derive significant resources from microorganism groups not considered in this study, such as bacteria, fungi and viruses. These are likely to be ingested by macroinvertebrates along with the abundant fine glacial rock fragments found in guts [78], [79], [87]. Alternatively, the highly patchy nature of the benthos in glacial rivers could mean that algal and/or invertebrate abundances were heavily under- or over-estimated, respectively, when sampling. When collecting macroinvertebrates by hand it was observed that the rocks with the most Chironomidae larvae had noticeable biofilm accumulations. Further work should be undertaken with stratified sampling techniques to ensure that the scaling coefficients reported here are not an artefact of spatial averaging from random sampling in this spatially heterogeneous environment.

Tritrophic food web statistics for the Odenwinkelkees web revealed a similar number of three node food chains to those in drought disturbed food webs of Woodward *et al*. [74] but far fewer than the equivalent webs from control treatments. Additionally, all of the statistics were lower than those from both control and drought treatment webs reported previously with the exception of link angles. Plots of U_upper_-A_between_ and two span-A_between_ for the Ödenwinkelkees web were within a similar constraint space of control webs from the Woodward *et al*. experiment and data for Tuesday Lake and the Ythan Estuary [72], [73], but their relatively narrow distribution was mostly in accordance with the webs constructed following drought disturbance. This narrowing may reflect similar underlying drivers of food web structure under severe habitat conditions, including a lack of larger bodied species, low community biomass and low production. In rivers where glacier retreat leads to a shift towards more benign habitat conditions over time, it can be expected that food webs will develop to display a wider range of *MN* scaling, particularly due to colonisation by larger organisms, and thus a more heterogeneous array of link angles will be evident.

### Conclusions and Areas for Further Research

Close relationships between individuals in the Ödenwinkelkees food web (i.e. short path lengths, relatively high connectance, short food chain lengths, constrained distribution of link angles) means that any factor affecting just one node can rapidly propagate throughout the web [66]. As glacial retreat is occurring in alpine environments worldwide, with associated increases in water temperature, channel stability, and the proportion of groundwater to stream flow, instream flora and fauna are consequently changing [14]. Warmer water temperature (e.g. downstream, or over time as glaciers retreat; [15], [18]) is linked strongly to the turnover of glacial stream community composition, with the introduction of previously unresolved species that compete with existing species for food sources. The introduction of species, either through an increased producer base [25] or the colonisation of additional invertebrates could quickly impact on food web structure. Further research to examine the nature of food web dynamics (in terms of connectance and size structure, as well as quantifying links) along the strong environmental gradients linked to glacier retreat would provide much needed information on the mechanisms of food web assembly as linked to successional processes [88], in addition to testing fundamental ideas related to how individuals, communities and ecosystems can be expected to respond to climate change [5], [6]. Some authors have suggested that climate change will lead to food webs being affected from the top down as larger organisms are affected disproportionately by environmental change [89], [90]. In glacial systems currently dominated by abundant small organisms, the opposite is likely as conditions ameliorate and larger taxa colonise.

Where invasions have occurred in streams with well-documented food webs, mean chain length, linkage density and connectivity all increased [62]; similar shifts in food web structure are likely with reduced glacial influence as more species colonise these communities (*cf*. [16]). However, if warming temperatures lead to the loss of cold-adapted species, which occur only in high altitude habitats, gamma diversity of species will decrease [9], [13], [16]. Alpine invertebrate species’ vulnerability is increased because their distributional range is limited to mountain ‘islands’ from which they cannot disperse to other areas. The potential impacts of such species losses need to be studied in the context of food web responses if we are to produce accurate predictions of ecosystem change.

## Supporting Information

Figure S1 **Yield effort curves constructed for each food web.**
(EPS)

Table S1 **Composite connectance food web data for the Ödenwinkelkees river (2006–2011).**
(DOCX)

Table S2 **Summary statistics for the 62 published webs and the four assembled for this study.**
(DOCX)
