# Supplementary material for: Food Web Structure in a Harsh Glacier-Fed River
Source: PLoS One. 2013 Apr 15;8(4):e60899. doi: 10.1371/journal.pone.0060899 (PMC3626691; doi:10.1371/journal.pone.0060899)
Supplement: Table S1 — Composite connectance food web data for the Ödenwinkelkees river (2006–2011). (DOCX) [file pone.0060899.s002.docx]

**Table S1:** Composite connectance food web data for the Ödenwinkelkees river (2006-2011)

|  | **Amorphous detritus** | ***Encyonema* sp.** | ***Gomphonema* sp. A** | ***Gomphonema* sp. B** | ***Diatoma mesodon*** | ***Meridian circulaire*** | ***Cymbella subaquealis*** | ***Achnathes* sp. A** | ***Achnanthes* sp. B** | ***Gomphonema/Cymbella*** | ***Fragilaria arcus*** | ***Fragilaria* sp.** | ***Diamesa latitarsis/steinboecki*** | **Orthocladiinae** |
| --- | --- | --- | --- | --- | --- | --- | --- | --- | --- | --- | --- | --- | --- | --- |
| ***Diamesa bertrami*** | 1 | 1 | 1 | 1 | 1 | 1 | 1 | 1 |  | 1 | 1 |  |  |  |
| ***Diamesa bertrami/latitarsis*** | 1 | 1 | 1 | 1 | 1 |  | 1 | 1 |  | 1 |  | 1 |  |  |
| ***Diamesa cinerella/zernyi*** | 1 | 1 | 1 |  | 1 | 1 | 1 | 1 |  | 1 |  |  |  |  |
| ***Diamesa dampfi/permacer*** | 1 | 1 | 1 | 1 | 1 |  | 1 | 1 | 1 | 1 |  |  |  |  |
| ***Diamesa latitarsis*** | 1 | 1 | 1 | 1 | 1 | 1 | 1 | 1 |  | 1 | 1 |  | 1 |  |
| ***Diamesa latitarsis/steinboecki*** | 1 | 1 | 1 | 1 | 1 | 1 | 1 | 1 |  | 1 | 1 |  | 1 |  |
| ***Pseudokiefferiella*** | 1 | 1 |  |  |  | 1 |  | 1 | 1 | 1 | 1 |  |  |  |
| **Orthocladiinae** | 1 |  |  |  |  |  |  |  |  |  |  |  |  |  |
| ***Prosimulium*** | 1 | 1 | 1 | 1 | 1 | 1 | 1 | 1 |  | 1 | 1 |  | 1 | 1 |
| ***Baetis* sp.** | 1 |  |  |  |  |  | 1 |  |  |  |  |  |  |  |
| ***Limnephilidae* juv.** | 1 | 1 |  |  |  |  | 1 | 1 |  | 1 |  |  |  |  |
