# Supplementary material for: Food Web Structure in a Harsh Glacier-Fed River
Source: PLoS One. 2013 Apr 15;8(4):e60899. doi: 10.1371/journal.pone.0060899 (PMC3626691; doi:10.1371/journal.pone.0060899)
Supplement: Table S2 — Summary statistics for the 62 published webs and the four assembled for this study. (DOCX) [file pone.0060899.s003.docx]

**Table S2:** Summary statistics for the 62 published webs and the four assembled for this study

| **Food Web** | **Reference** | **Location** | **S** | **L** | **L/S** | **C** | **D** | **MCL** |
| --- | --- | --- | --- | --- | --- | --- | --- | --- |
| **2006** |  | Ödenwinkelkees | 13 | 16 | 1.23 | 0.05 | 2.26 | 2.00 |
| **2008** |  | Ödenwinkelkees | 19 | 51 | 2.68 | 0.14 | 1.95 | 2.36 |
| **2011** |  | Ödenwinkelkees | 19 | 67 | 3.53 | 0.19 | 1.76 | 2.32 |
| **Composite web** |  | Ödenwinkelkees | 23 | 85 | 3.70 | 0.16 | 1.86 | 2.28 |
| **Lavandier and Decamps. (1983)** | [31] | French Pyrénées (n=3) | 16-30 | 46-93 | 2.88-3.72 | 0.10-0.18 |  |  |
| **Hildrew et al. (1985)** | [91] | Broadstone Stream, UK | 24 | 90^□^ | 3.75 | 0.16 |  |  |
| **Lancaster and Roberston. (1995)** | [92] | Broadstone Stream, UK | 33 | 122 | 3.70 | 0.11 |  |  |
| **Tavares-Cromar and Williams. (1996)** | [93] | Duffin Creek, Canada, (n=7) | 31-39 | 101-146 | 3.14-3.74 | 0.09-0.11 |  |  |
| **Jaarsma et al. (1998)*** | [27] | Healy Creek, New Zealand | 96 | 589 | 6.14 | 0.14 |  | 3.02 |
| **Jaarsma et al. (1998)*** | [27] | Dempsters Creek, NZ | 107 | 967 | 9.04 | 0.19 |  | 4.67 |
| **Townsend et al. (1998)** | [1] | South Island, NZ (n=10) | 86-113 | 353-966^▼^ | 4.31-9.03 | 0.04-0.08 | 2.27-2.31^▲^ | 1.79-4.42 |
| **Hall et al. (2000)** | [28] | Appalachian Mountains | 35 | 200 | 5.71 | 0.16 |  |  |
| **Woodward and Hildrew.( 2001)** | [62] | Broadstone Stream, UK (n=4) | 24-34 | 109-170 | 4.42-5.12 | 0.13-21 |  | 4.88-5.38 |
| **Schmid-Araya et al. (2002)** | [94] | Broadstone Stream, UK (n=5) | 54-128 | 229-721 | 4.09-5.63 | 0.04-0.08 |  |  |
| **Thompson and Townsend. (2003)** | [29] | Southeast USA (n=4) | 71-105 | 126-343 | 2.08-3.27 | 0.07-0.08 |  | 1.52-3.13 |
| **Thompson and Townsend. (2003)** | [29] | South Island, New Zealand (n=6) | 69-92 | 190-626 | 2.75-6.42 | 0.08-0.18 |  | 1.68-2.35 |
| **Mantel et al. (2004)** | [95] | Tai Po Kau Forest, Hong Kong | 28 | 157 | 5.61 | 0.20 |  | 2.04 |
| **Thompson and Townsend. (2005)**** | [96] | South Island, NZ (n=4) | 49-79 | 110-240 | 2.18-3.04 | 0.03-0.05 |  | 1.56-2.12 |
| **Woodward et al. (2008)** | [97] | Bere Stream, UK | 142 | 1383 | 9.74 | 0.07 |  |  |
| **Hernandez and Sukhdeo (2008)** | [98] | Muskingham Brook, USA (n=8; “parasite free web”) | 26-39 | 62-123 | 2.38-3.15 | 0.08-0.10 |  | 2.86^●^ |
| **Layer et al. (2010)^□^** | [40] | UK streams (n=20) | 19-87 | 56-1653 | 2.55-19.0 | 0.12-0.29 |  |  |
| **Brown et al. (2011)** | [30] | Mill stream, UK (n=4) | 61-71 | 320-492 | 5.25-7.34 | 0.08-0.11 | 2.03-2.17 | 2.06-2.16 |

*Maximum food web **data only for the four streams not included in [1] and [29] ^□^cited by Brown *et al* [30] ^▼^from Thompson and Townsend [96] ^▲^from Dunne *et al* [67] ^●^value from a composite food web

**References^1^**

91. Hildrew AG, Townsend CR, Hasham A (1985) The predatory Chironomidae of an iron-rich stream: feeding ecology and food web structure. Ecological Entomology 10: 403-413.

92. Lancaster J, Robertson AL (1995) Microcrustacean prey and macroinvertebrate predators in a stream food web. Freshwater Biology 34: 123-134.

93. Tavares-Cromar AF, Williams DD (1996) The importance of temporal resolution in food web analysis: evidence from a detritus-based stream. Ecological Monographs 66: 91-113.

94. Schmid-Araya JM, Schmid PE, Robertson A, Winterbottom J, Gjerløv C, et al. (2002) Connectance in stream food webs. Journal of Animal Ecology 71: 1056-1062.

95. Mantel SK, Salas MD (2004) Food web structure in a tropical Asian forest stream. Journal of the North American Benthological Society 23: 728-755.

96. Thompson RM, Townsend CR (2005) Food-web topology varies with spatial scale in a patchy environment. Ecology 86: 1916-1925.

97. Woodward G, Papantoniou G, Edwards F, Lauridsen RB (2008) Trophic trickles and cascades in a complex food web: impacts of a keystone predator on stream community structure and processes. Oikos 117: 683-692.

98. Hernandez AD, Sukhdeo MVK (2008) Parasites alter the topology of a stream food web across seasons. Oecologia 156: 613-624.

^1^References not listed in the main text
